# Retraction Note: DNA encoding schemes herald a new age in cybersecurity for safeguarding digital assets

**DOI:** 10.1038/s41598-025-88627-8

**Published:** 2025-02-11

**Authors:** Sehrish Aqeel, Sajid Ullah Khan, Adnan Shahid Khan, Meshal Alharbi, Sajid Shah, Mohammed EL Affendi, Naveed Ahmad

**Affiliations:** 1 Faculty of Computer Science and Information Technology, Universiti Malaysia Sarawak, 94300 Kota Samarahan, Malaysia; 2 Department of Information Systems, College of Computer Engineering and Sciences, Prince Sattam Bin Abdulaziz University, AlKharj, Kingdom of Saudi Arabia; 3 Department of Computer Science, College of Computer Engineering and Sciences, Prince Sattam Bin Abdulaziz University, Al-Kharj, Saudi Arabia; 4 EIAS Lab, CCIS, Prince Sultan University, Riyadh, Saudi Arabia; 5 College of Computer Information Sciences, CCIS, Prince Sultan University, Riyadh, Saudi Arabia

Retraction of: *Scientific Reports* 10.1038/s41598-024-64419-4, published online 15 June 2024

The Editors have retracted this Article.

After the publication of the Article, Editors were alerted to numerous concerns regarding this paper, including the presence of nonsensical phrases, non-existing references (80-90), the mismatch between text & quoted references (e.g., references 19-20, 35, 44-46) and incorrect labelling in Figure 5 (Thymine is listed twice). The authors were contacted for an explanation of concerns and were unable to provide a satisfactory response. The Editors therefore have lost confidence in the reliability of the content presented in this Article.

Sajid Ullah Khan disagrees with the retraction. Adnan Shahid Khan did not explicitly state if they agree or disagree with the retraction. The Editors were not able to obtain the current contact details of Mohammed EL Affendi. Sehrish Aqeel, Meshal Alharbi, Sajid Shah, and Naveed Ahmad have not responded to correspondence from the Editors about this retraction.

